# Bilayer Hydrogels for Wound Dressing and Tissue Engineering

**DOI:** 10.3390/polym14153135

**Published:** 2022-08-01

**Authors:** Olga Luneva, Roman Olekhnovich, Mayya Uspenskaya

**Affiliations:** Center of Chemical Engineering, ITMO University, Kronverkskiy Prospekt, 49, bldg. A, 197101 Saint-Petersburg, Russia; r.o.olekhnovich@itmo.ru (R.O.); mv_uspenskaya@itmo.ru (M.U.)

**Keywords:** bilayer hydrogel, wound dressing, wound healing, skin sensors, in vivo experiments, drug release

## Abstract

A large number of different skin diseases such as hits, acute, and chronic wounds dictate the search for alternative and effective treatment options. The wound healing process requires a complex approach, the key step of which is the choice of a dressing with controlled properties. Hydrogel-based scaffolds can serve as a unique class of wound dressings. Presented on the commercial market, hydrogel wound dressings are not found among proposals for specific cases and have a number of disadvantages—toxicity, allergenicity, and mechanical instability. Bilayer dressings are attracting great attention, which can be combined with multifunctional properties, high criteria for an ideal wound dressing (antimicrobial properties, adhesion and hemostasis, anti-inflammatory and antioxidant effects), drug delivery, self-healing, stimulus manifestation, and conductivity, depending on the preparation and purpose. In addition, advances in stem cell biology and biomaterials have enabled the design of hydrogel materials for skin tissue engineering. To improve the heterogeneity of the cell environment, it is possible to use two-layer functional gradient hydrogels. This review summarizes the methods and application advantages of bilayer dressings in wound treatment and skin tissue regeneration. Bilayered hydrogels based on natural as well as synthetic polymers are presented. The results of the in vitro and in vivo experiments and drug release are also discussed.

## 1. Introduction

Skin, as the largest organ of the human body, protects the body from undesirable environmental influences, preventing the penetration of contaminants and the development of infections [1]. In addition, the skin plays an important role in homeostasis, thermoregulation, and immune response [2].

In the framework of the modern concept, all lesions of the skin resulting from physicochemical or thermal effects are called wounds, which are divided into acute and chronic wounds [3,4]. Acute wounds are skin lesions that require healing within 8–12 weeks, for example, burns and chemical injuries. A chronic wound is defined as a wound characterized by a long healing process, up to several months, followed by scarring [5]. At the same time, skin and soft tissue infections slow down the healing process and can lead to life-threatening conditions, thereby increasing morbidity and mortality [6].

The complex of local and general reactions of the body, occurring from the moment of wound formation to its healing, is called the wound healing process [7]. There are three phases of the wound process:

- Inflammation (substrate). At the moment of damage, blood enters the wound, bringing into it not only cellular elements, but also various proteins, among which fibrinogen is of the greatest importance.
- Proliferation (regeneration). The wound is filled with a cellular matrix, the basis for scar formation, and is reduced. Continues from 5 days up to 3 weeks after the injury. During this period, there is a proliferation of tissue.
- Maturation (remodeling).

The treatment consists of the timely change of dressings on wounds to accelerate the healing of damaged areas, in order to avoid complications and scarring as well as infection by pathogenic microorganisms. Modern wound dressings include various groups such as hydrogels [8], hydrocolloids [9], alginates [10], synthetic foam dressings [11], and vapor-permeable films [12]. The criteria in choosing the optimal dressing are based on its ability to support the healing of the skin wound [13]: (a) maintaining a moist environment; (b) increased migration of epidermal cells; (c) promotion of the synthesis of tissue; (d) ensuring optimal air and vapor permeability between the damaged tissue and the environment; (e) maintaining an appropriate tissue temperature to improve blood flow; (f) protection against bacterial infection; (g) optimal adhesion to the wound and easy removal after the healing; and (h) sterility, non-toxicity, and hypo allergenicity.

Compared to other types of synthetic wound dressings, hydrogels are three-dimensional (3D) networks consisting of physically or chemically cross-linked hydrophilic polymers, which are the best choice as a dressing material [14,15]. Hydrogels contain a volume of liquid that exceeds their own by hundreds of times. Due to the high water content, these materials have sufficient biocompatibility and can be obtained with a water content very similar to that of biological tissues (70%) or much higher (up to 99% of water). With such structures, hydrogels are able to swell without the dissolving of the polymer, which gives them characteristics similar to those of soft tissues. By maintaining a moist wound environment, hydrogel dressings protect it from inflammation and bacterial growth, creating a physical barrier to protect the wound from microbial invasion, infection, and supporting fibroblast proliferation and keratinocyte migration. These processes are necessary for epithelialization and the acceleration of wound healing [16,17,18]. The process of removing such dressings becomes less painful, since the coating does not “dry” to the wound surface. Hydrophilic groups in the polymer chain of hydrogels, such as -COOH, -SO_3_H, -NH_2_, and -OH increase the ability of hydrogels to absorb water [19]. Synthetic polymers or natural polymers can serve as materials for hydrogel dressings. The polymers used have to be biodegradable, biocompatible, non-toxic, non-mutagenic, and non-immunogenic [20]. 

Synthetic polymers can be obtained through the polymerization of monomers. The advantage of synthetic polymers is the reproducibility of properties and the regularity of the macromolecular chain [21,22,23,24,25,26,27,28] (Table 1).

Natural polymers are usually polysaccharides and proteins consisting of glycosidic and amino acid repeating units [36,37,38,39,40,41,42,43,44] (Table 2). Natural polymers have a number of advantages such as the ability to imitate the tissue of the recipient and to bind to biological systems, metabolic compatibility, non-toxicity and weak inflammatory reactions, and the degradation by enzymes as well as the further use of the degradation products in cellular metabolism. However, the disadvantage of natural polymers is their low mechanical stability.

**Figure 1:**
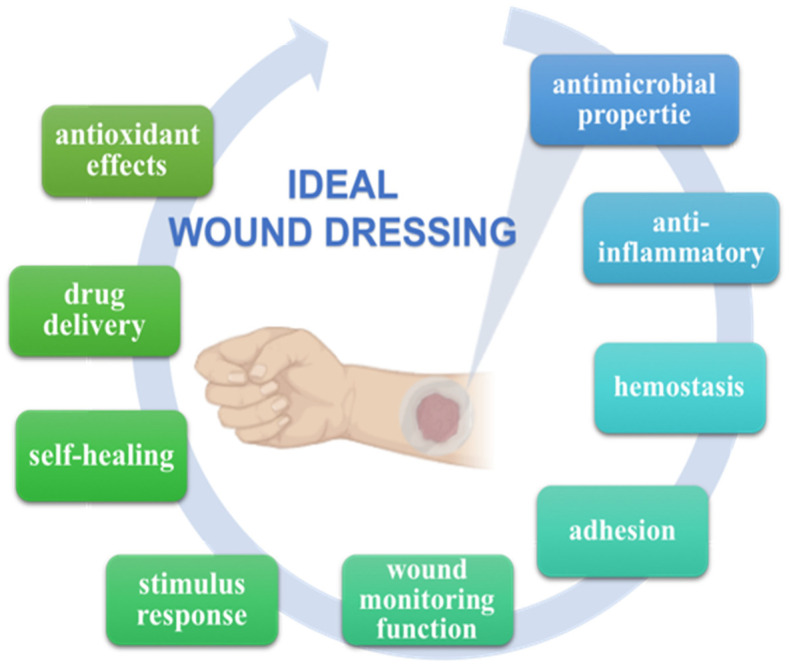
The main criteria for an ideal wound dressing. Created with the use of Biorender.com.

Hydrogel dressings demonstrate advanced functions in the wound process such as antimicrobial properties, adhesion and hemostasis, anti-inflammatory and antioxidant effects, drug delivery, self-healing, stimulus response, and conduction [45,46] (Figure 1). 

A mesh structure protects the wound from the infection and prevents the penetration of microorganisms into the wound area and the formation of inflammatory processes [47]. 

In addition, it is possible to graft hydrogel scaffolds of various structures and cells on a three-dimensional network, which have a therapeutic effect and accelerate healing [48,49,50]. The structure of hydrogels allows for the transport of bioactive molecules such as antibiotics or natural biologically active compounds to the wound [51,52].

Hydrogels are a promising type of material that have already entered into the commercial market with successful performance indicators [53]. Thus, the effect of the NanoDOX^®^ hydrogel with an antibiotic compared with the placebo hydrogel on incised surgical wounds was evaluated. Absolute wound healing was observed in all volunteers [54]. Studies of the hydrogel material with the NanoDOX^®^ brand antibiotic for atopic dermatitis observed the decrease in pathogenic flora in half of the participants; the others did not notice its growth at all. However, ongoing studies on the effectiveness of a number of commercial hydrogel materials have shown related results and revealed the presence of shortcomings [55].

**Table 2:** Natural polymer systems for wound dressing.

Polymer Basis	Hydrogel Composition	Key Effects	Ref.
COL	COL-HA	ECM mimic; promoting fast spontaneous wound healing	[56]
	HLC-HA-CCS	Non-toxic, biocompatible with promoting wound cell proliferation and burn wound healing	[57]
	COL-CS	Self-healing capacity, injectability, antibacterial ability; promoting the wound healing, hemostatic ability; sensitive epidermal sensoring;	[58]
	AC-OSA-PB	Antimicrobial activity; promotion cell proliferation and migration, angiogenesis accelerating	[59]
	gellan gum−COL	Inflammation reducing; promoting complete skin regeneration; mechanical stability	[60]
GA	konjac/FG -matrine	High elasticity, enhancing the blood compatibility and antibacterial activity	[61]
	gellan-GA-TA	Injectable, shear-thinning and self-recovery; antimicrobial activity, accelerating healing	[62]
	GA-HA-CNC	Attachment, growth, proliferation of fibroblasts, skin regeneration, mechanical functionality	[63]
	GelMA- ZIF-8 nanoparticles loaded double enzyme system	Sprayable; anti-inflammatory, hypoglycemic and antibacterial; promotion diabetic wound healing with scar-free.	[64]
	GA-TA	Mechanical strength, antibacterial activity, water vapor and oxygen permeable	[65]
CS	lignin–CS– PVA	Bactericidal activity, high mechanical strength, large tensile deformation	[66]
	CS-PEC-LDC	Thermosensitive; self-adhesion to skin	[67]
	DLs-CS	Targeting system for the treatment of chronic wounds	[68]
CL	BC-AA loaded with HEKs and HDFs	Acceleration of burn wound healing	[69]
	CMC-SF-Mg(OH)_2_NPS	Enhanced mechanical strength, excellent hemolysis response, bactericidal activity	[70]
	CMC-PEG	Assisting skin wound healing and regeneration;	[71]
Alg	TOBC/CA-Zn^2+^	Maintain mechanical properties, antibacterial properties; cell adhesion and proliferation;	[72]
	Alg/Vit D_3_	Induced cells proliferation and the highest cell growth; accelerate wound healing	[73]
	Alg/Nar	Accelerated healing of excisional wound	[74]

COL—collagen, HA—hyaluronic-acid, ECM—extracellular matrix, CCS—carboxylated chitosan, HLC—human-like collagen, CS—chitosan, AC—aminated collagen, OSA—oxidized sodium alginate, PB—antimicrobial peptides (polymyxin B sulfate and bacitracin), GA—gelatin, FG—fish gelatin, TA—tannic acid, CNC—cellulose nanocrystals, GelMA—methacrylic anhydride-modified gelatin, ZIF-8—zeolitic imidazolate frameworks, PVA—polyvinyl alcohol, PEC—pectin, LDC—lidocaine hydrochloride, DLs—curcumin-containing deformable liposomes, BC—bacterial cellulose, AA—acrylic acid, HEKs—human epidermal keratinocytes, HDFs—human epidermal keratinocytes, CL—cellulose, CMC—carboxymethyl cellulose, SF—silk fibroin, Mg(OH)_2_NPS—magnesium hydroxide nanoparticles, PEG—polyethylene glycol, TOBC—2,2,6,6-tetramethylpiperidine-1-oxyl oxidized bacterial cellulose, ALg—alginate, CA—calcium alginate, Nar—naringenin.

**Figure 2:**
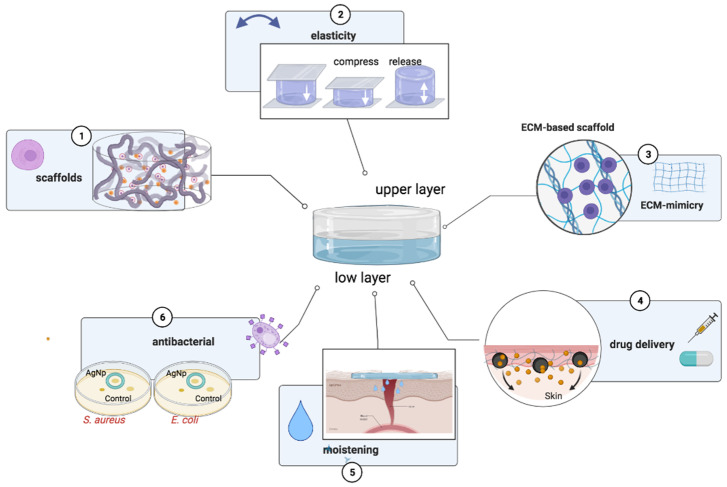
The application advantages of bilayer dressings in wound treatment and skin tissue regeneration. Created with the use of Biorender.com.

Bilayer hydrogel dressings, due to their structure and variety of preparation methods, have the ability to combine the above-mentioned properties. Thus, by meeting the criteria for an ideal wound dressing, these biomaterials have found wide application in wound healing and skin tissue engineering. This review summarizes the methods and application advantages of bilayer dressings for wound treatment and skin tissue regeneration (Figure 2). Additionally, the in vitro and in vivo effects are considered. 

## 2. Biomedical Application of Bilayer Hydrogels

Bilayer hydrogel materials have a number of multifunctional properties that allow them to be widely used in biomedicine, for example, in drug delivery systems, obtained by UV copolymerization and the cross-linking of N-isopropylacrylamide (NIPAm) and acrylamide (AAm) monomers [75,76,77,78]; in the treatment of osteochondrosis as a biomimetic that provides a microenvironment for the maintenance and attachment of cells and viability [79]; or as two-layer scaffolds for ligament regeneration based on chitosan–hyaluronic acid with a hydrogel coating of poly(caprolactone) [80]. One of the interesting directions for the use of bilayer hydrogels in biomedicine is the creation of actuators [81]. Due to the presence of a bilayer structure, the distribution of variable stimulus responses into two layers, hydrogel actuators can programmatically change their shape and perform mechanical activity by changing the humidity and pH [82,83,84]. The fabrication of a bilayer structure is an efficient way to obtain an anisotropic hydrogel [85]. One of the reasons for the functioning of a bilayer hydrogel actuator is the different sorption capacity of individual layers with the encapsulation of the thermosensitive polymer—poly(n-isopropylacrylamide) [86,87].

### 2.1. Bilayer Hydrogels for Skin Regeneration

A separate area is occupied by bilayer hydrogel dressings for the treatment of skin diseases. The biocompatibility of hydrogels is explained by their similarity to the macromolecular components of the body. An optimal hydrogel scaffold should have high degradation rates as well as reproducible and controllable characteristics such as (i) material composition; (ii) environment; (iii) structure; (iv) surface treatment; (v) external intervention; and (vi) physical loading, so that it can be used in wound healing, cell differentiation, angiogenesis, etc. [88,89]. 

Bilayer hydrogels have revealed greater biocompatibility with living organisms. In addition, the two layers, showing multifunctional properties, have different tasks: the upper layer protects against the influence of external factors and maintains a humid environment, while the lower layer promotes cell adhesion and proliferation [90].

Skin lesions of a diverse nature of origin increase the risk of consequences for the body, even after years. In order to avoid the appearance of adhesions after surgery, a bilayer hydrogel dressing was developed consisting of gelatin and polyvinyl alcohol (PVA) with simultaneous non-reagent radiation-induced crosslinking by γ-rays and having both dressing and anti-adhesion properties [91]. 

Wounds caused by diabetes differ from typical skin lesions and healing stages [92]. In diabetic wounds, there are changes in keratinocyte function that occur due to the effect of insulin on the proliferation, differentiation, and migration of keratinocytes, which consequently leads to delayed wound healing [93]. Combining approaches for the treatment of diabetic wounds have been embodied by the creation of bilayer wound dressings with different therapeutic properties [94]. Gelatine methacrylamide (GelMa) was used as the base for the hydrogel double layer dressing. In addition, referring to previous studies, in order to impart and combine therapeutic properties in each of the layers, the authors of the work encapsulated the following additives: the top layer consisted of a layer of ε-poly-L-lysine (PLL) with strong antibacterial properties against infections and methacryloyl-substituted recombinant tropoelastin (MeTro), which helps to repair the wound and increase the rate of wound healing [95,96]. The low layer of the dressing with the addition of VEGF-mimetic peptides, which can enhance the angiogenesis process and collagen fiber regeneration, is porous. Initially, the two layers were injectable, but after exposure to blue light (405 nm) for 3 min, the liquid solidified on the wound without changing shape.

**Figure 3:**
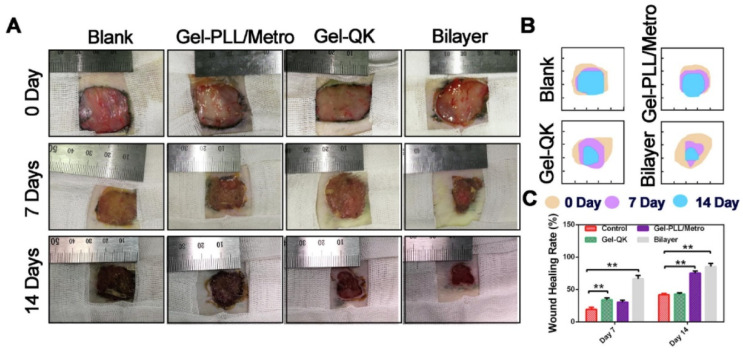
The in vivo evaluation of wound healing after treatment with different hydrogels. (**A**) Wound images from the four individual groups (blank control, Gel-PLL/MeTro, Gel-QK, and bilayer) at 0, 7, and 14 days. (**B**) Traces of wound closure. (**C**) Statistical results of the wound healing rates. ** *p* < 0.01. Reproduced from [94], with permission from Elsevier, 2022.

An in vivo study has proven the effectiveness of the use of a two-layer dressing, which deprives all of the necessary therapeutic methods for the treatment of a diabetic wound in comparison with single-layer component hydrogels (Figure 3).

**Figure 4:**
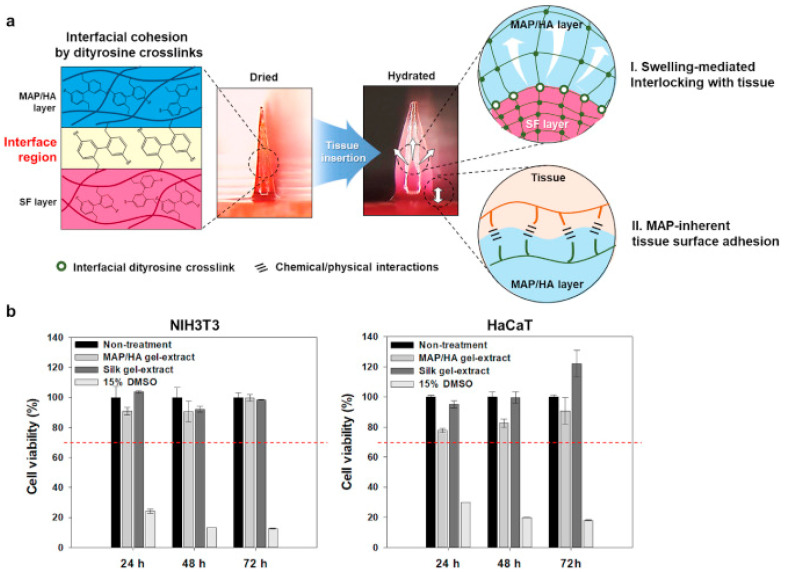
A schematic illustration of a hydrogel-forming, double-layered, adhesive MN patch and the cytocompatibility of the light-crosslinked hydrogels. (**a**) A schematic illustration for the proposed working mechanisms of a hydrogel-forming adhesive MN patch consisting of a MAP-based swellable and sticky shell and a SF-based non-swellable core. After tissue insertion, the MN patch could achieve adhesion on wet and/or dynamic biological tissues via both the MAP-derived surface adhesive and swelling-mediated physical interlocking properties. (**b**) In vitro cytocompatibility evaluation of each MAP/HA and SF gel-extract using NIH3T3 fibroblasts and HaCaT keratinocytes (*n* ≥ 3). The red dashed line indicates a cytotoxic effect that is considered as a reduction in cell viability by more than 30% (for the interpretation of the references to color in this figure legend, the reader is referred to the web version of this article). Reproduced from [99], with permission from Elsevier, 2022.

The sorption capacity of hydrogels is an important material parameter that determines their unique properties and drug release control from polymer networks [97]. Hydrogels, as three-dimensional polymeric networks, can be chemically stable and able to reversibly swell and hold a large volume of liquid [98]. A bioinspired hydrogel-forming scaffold was developed as a two-layer microneedle (MN) adhesive, consisting of a swelling shell based on mussel adhesive protein/hyaluronic acid (MAP/HA) and a non-swelling core based on silk fibroin (SF) with silkworm cocoon, the action of which is based on its use mainly in a humid environment [99]. Endoparasites tightly fixed in the host’s intestines have also been investigated. The results of the ex vivo study showed that the resulting hydrogel composition had excellent wound sealing ability against luminal leaks (139.7 ± 14.1 mm Hg), which was comparable to the suturing (151.0 ± 23.3 mm Hg). The authors, referring to previous studies, stated that MAPs, which contain almost equal amounts of both aromatic and cationic residues, can efficiently attach to biological substrates through various chemical/physical interactions such as the cation–π interaction, π–π-stacking, hydrogen bonds, and electrostatic interactions [100,101,102]. At the same time, the two-layer coating demonstrates adhesion to both semi-dry and wet skin, unlike a commercial medical plaster, when it was applied only to semi-dry surfaces. Drug delivery can be quite effective due to the excellent interfacial adhesion via various physical/chemical interactions (Figure 4).

#### Antibacterial and Antioxidant Activity

The antimicrobial activity of hydrogels may be mediated either by encapsulation in the gel network and the subsequent release of drugs, or some gels are antimicrobial in the case when the hydrogel itself is polycationic and the network also exhibits antibacterial activity itself [103,104]. The choice of the antimicrobial component depends on the virulence, the type of patient and pathologies, the type and location of the wound, and the amount of exudate produced by the wound [105]. Antibacterial drugs include antibiotics, some biological extracts, natural polymers, and some metal nanoparticles [106]. Recently, hydrogels with sensitive components such as pH and are light- and temperature-sensitive have attracted scientific and practical interest. 

Oustadi et al. developed a locally releasing ibuprofen bilayer dressing with membrane bactericidal activity. Initially, the layer with 1% ibuprofen was obtained in various ratios of a poly(vinyl alcohol) (PVA)/poly(vinyl pyrrolidone) (PVP) solution. The hydrogels were crosslinked using the repeated freeze–thaw cycles followed by lyophilization as the bottom layer [107]. 

The loading efficiency and loading capacity of the ibuprofen of 88.50 ± 2.05% and 0.087 ± 0.003%, respectively, were achieved. The drug release percentage was 30% and 50% at the first and third hours, respectively. These results indicate that at the initial stage, the drug was burst released. The final release was approximately 90% and 100% after 10 and 15 h, respectively. The drug was completely released within 16 h, which underlines the useful property of antibacterial materials to prevent bacterial growth at the early stages of the wound process.

The study of the hydrogel mechanical properties revealed the decrease in the tensile strength with the increase in the PVP content. The authors suggest that the annular structure of PVP plays a role in this phenomenon. PVP creates more space between the polymer chains for greater mobility, and as a result, as the PVP content increases, the structure becomes less dense, resulting in the decrease in PVA/PVP tensile strength.

Various skin diseases require treatment with antibiotics. The aim of the research by Tamahkar et al. was the creation of a multilayer wound dressing with the controlled release of antibiotics [108]. Hydrogels were prepared in four layers using carboxylated polyvinyl alcohol (PVA-C), gelatin (G), hyaluronic acid (HA), and gelatin, respectively, via a layer-by-layer self-assembly technique. The top layers (PVA-C and G) provided moisture control and a physical barrier to microorganisms. The HA-based middle layer was designed as the antibiotic loaded layer. The lower layer served as the control membrane for the release of the antibiotic and ensured the removal of excess exudate from the wound. The following models were used to describe the release of the antibiotic from the hydrogel matrix: Zero-order model (1); First-order model (2); Higuchi model (3); and the Korsmeyer–Peppas model (4) (Table 3).

Q_t_ = Q_0_ + *k*_0_*t* (1)

lnQ_t_ = lnQ_0_ − *k*_1_*t* (2)

(3) $$Qt_{\;}=k_{H}\surd t$$

(4) $$\frac{Q_{t}}{Q_{\mathrm{eq}}\;}=k_{\mathrm{KP}}t^{n}$$

where Q_t_ is the drug released amount at time *t*; Q_0_ is the initial drug amount of the release medium; Q_eq_ is the amount of drug released at equilibrium; *k*_0_ is the rate constant of the zero order model; *k*_1_ is the rate constant of the first order model; *k*_H_ the rate constant of the Higuchi kinetic model; *k*_KP_ is the rate constant of the Korsmeyer–Peppas kinetic model; *t* is the release time; *n* is the release exponent.

The zero-order kinetic model was the most adequate parameter to describe the drug release because the drug release was only time dependent and independent from the drug concentration. Nearly 65% of ampicillin was released within 7 days, indicating that the long-term release profile could be achieved using the prepared multilayer hydrogels. The authors of the study developed the hydrogel dressing with prolonged time periods of drug release compared with the previously fast releasing kinetics in drug release studies [109,110,111,112].

The programmed release of an antibiotic over a specified period has been demonstrated by Contardi et al. Wound dressing production is an environmentally friendly water-based molding process where the first layer (for the direct contact with the wound) is polyvinylpyrrolidone (PVP) containing the commercial antiseptic Neomercurocromo^®^ (Neo), and the second layer is a mixture of hyaluronic acid (HA) and PVP containing ciprofloxacin [113]. In vitro drug release measurements were collected from the following samples: ciprofloxacin and eosin release from BL (full bilayer), eosin release from BLN (bilayer without ciprofloxacin), ciprofloxacin release from SLHC (lower layer only), and eosin release from SLHC (lower layer only). 

**Figure 5:**
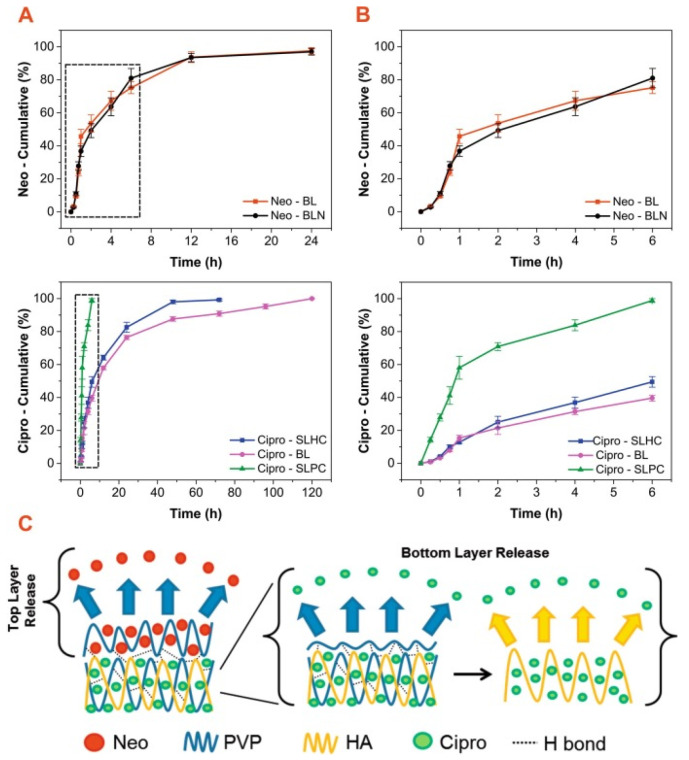
(**A**) The release profiles of eosin (**top** panels) and ciprofloxacin (**bottom** panels) over a period of 24 and 120 h, respectively. (**B**) The release results of eosin (**top** panels) and ciprofloxacin (**bottom** panels) for the first 6 h. (**C**) A schematic representation of the hypothetical drug release mechanism from the bilayer construct placed on a moist medium such as a wound. The potential establishment of hydrogen bonds within the top layer and at the interface between the top and bottom layers are shown as dashed lines. Reproduced from [113], with permission from Elsevier, 2022.

Glycerin was used as the plasticizer. Peel adhesion tests showed self-adhesion to both wet and dry human skin surfaces. This material showed high antibacterial activity against *Staphylococcus aureus*, *Escherichia coli,* and *Pseudomonas aeruginosa* and reliable adhesion (Figure 5).

The presence of silver nanoparticles (AgNP) endows the bilayer hydrogel with antibacterial properties and promotes an accelerated healing process. 

**Figure 6:**
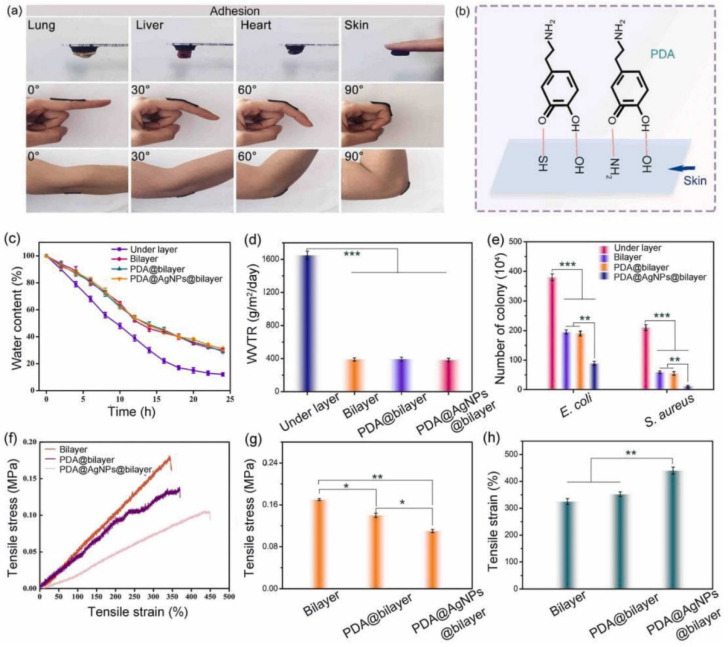
(**a**) The adhesiveness of the PDA@AgNPs@bilayer hydrogels and the(**b**) adhesion mechanism. (**c**) The moisture retention ability, (**d**) WVTR, (**e**) bacterial penetration numbers, and (**f**–**h**) mechanical properties of the hydrogels. Reproduced from [114], with permission from Elsevier, 2022.

The top layer of the material consists of carboxylated chitosan, which, due to its bacterial activity, is able to prevent the penetration of a bacterial infection. The basis of the bottom layer is polyvinyl alcohol (PVA) and polyethylene glycol (PEG) as a blowing agent. A layer with small pores is able to maintain a moist environment, and a layer with large pores absorbs the wound exudate and provides oxygen exchange. This material also exhibits high adhesive activity due to the catechin groups of PDA [114] (Figure 6).

**Figure 7:**
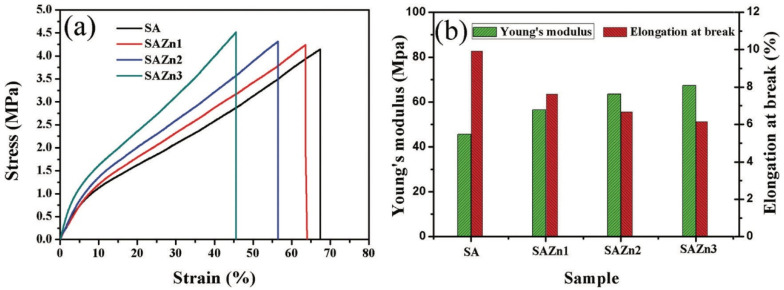
(**a**) The tensile stress–strain curves, and (**b**) the Young’s modulus and elongation at break of the bilayered hydrogel films. Reproduced from [115], with permission from the Royal Society of Chemistry, 2022.

Another option for the use of alginate salts in obtaining an antibacterial two-layer dressing was described in [115]. Zinc oxide (ZnO) nanoparticles have also been used as an antibacterial agent. ZnO shows significant growth inhibition of a wide range of bacteria [116]. Hydrogels, in which metal oxide nanoparticles are encapsulated, also have good antibacterial properties, but unlike metal nanoparticles, the antibacterial mechanism of metal oxide nanoparticles is different [117]. There are two common antibacterial mechanisms of the action of metal oxide nanoparticles in hydrogel materials: (1) antimicrobial toxicity arises from the production of metal ions by the nanoparticles; and (2) oxidative stress due to the formation of reactive oxygen species (ROS) on the surface of nanoparticles [118]. The outer layer of the bilayer hydrogel material of the ZnO/SA film prevented the entry of bacteria, while the authors reported on the ability of oxygen to diffuse and maintain a favorable moist environment at the wound boundary; the inner layer was water-absorbent for the wound exudate. It is worth noting that the most well-known theory of alginate–metal binding is the “Egg-box” model. This model assumes the arrangement of the hydroxyl groups of polymeric α-L-guluronates (G) in such a way that special cavities for cations are formed, like an egg carton for eggs [119]. The zinc oxide nanoparticle encapsulation not only led to the manifestation of antibacterial properties and an increase in the healing rate, but also improved the mechanical properties of the resulting material—the Young’s modulus increased by 10%, and the elongation at break decreased from 67.4 to 45.6%, respectively (Figure 7) (Table 4).

As a wound dressing for skin and the prevention soft tissue infections, a hybrid based on an antibacterial bilayer sponge scaffold made of silk fibroin/gelatin (SF/Gel) loaded with various concentrations of a cationic antimicrobial peptide (CM_11_ peptide) was developed. The release of the CM_11_ peptide after 500 min was observed from the samples, while the complete release of up to 80% was observed after 4000 min. The authors confirmed that compared to the blank (SF/Gel), the material modified with antimicrobial peptides demonstrated improved mechanical and antibacterial properties against *Staphylococcus aureus, Escherichia coli,* and *Pseudomonas aeruginosa* [120]. 

**Figure 8:**
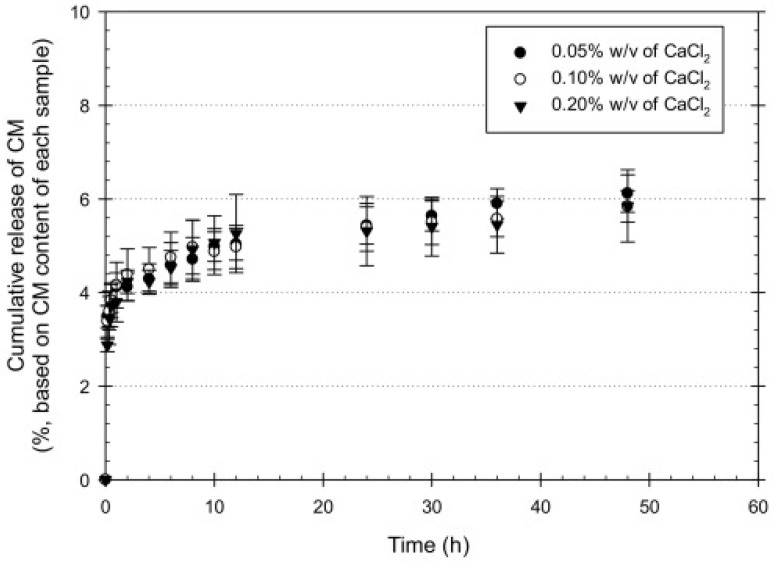
The cumulative release profiles of CM from CMx-loaded SA hydrogels (*n* = 5). Reproduced from [122], with permission from Elsevier, 2022.

In addition, the polymers used have to be able to provide adequate mechanical support and provide an environment for cell adhesion, proliferation, and differentiation [121]. The creation of bilayer systems allows for the use of polymers with mutually complementary properties. Sodium alginate is biocompatible, non-toxic, economically available, but at the same time cannot prevent bacteria from entering into the wound. Kiti et al. obtained a two-layer structure containing sodium alginate with the addition of curcumin to accelerate wound healing and a separate layer of chitosan, which provided the necessary mechanical support and adhesion of the dressing to the skin, and also slowed down the growth of bacteria due to its antibacterial activity [122]. Calcium chloride was used as the crosslinking agent, which improved the mechanical properties, and the results of the tensile tests showed that an increase in CaCl_2_ led to an increase in the mechanical properties, but at the same time, moisture absorption, water swelling, and weight loss decreased. At the same time, the samples with the lowest content of the cross-linking agent showed the highest release rate (Figure 8).

The release kinetics of CM from the CMx-loaded SA layer were characterized according to the following equation:

(5) $$\frac{M_{t}}{M_{\infty }}=kt^{n}\mathrm{for}\;\frac{M_{t}}{M_{\infty }}<0.6$$

where *M_t_* is the cumulative amount of the CM released at an arbitrary time *t*; *M_∞_* is the cumulative amount of the CM released at an infinite time; *n* is an exponent characterizing the mechanism; and *k* is the release rate of CM incorporated in the system (Table 5).

**Figure 9:**
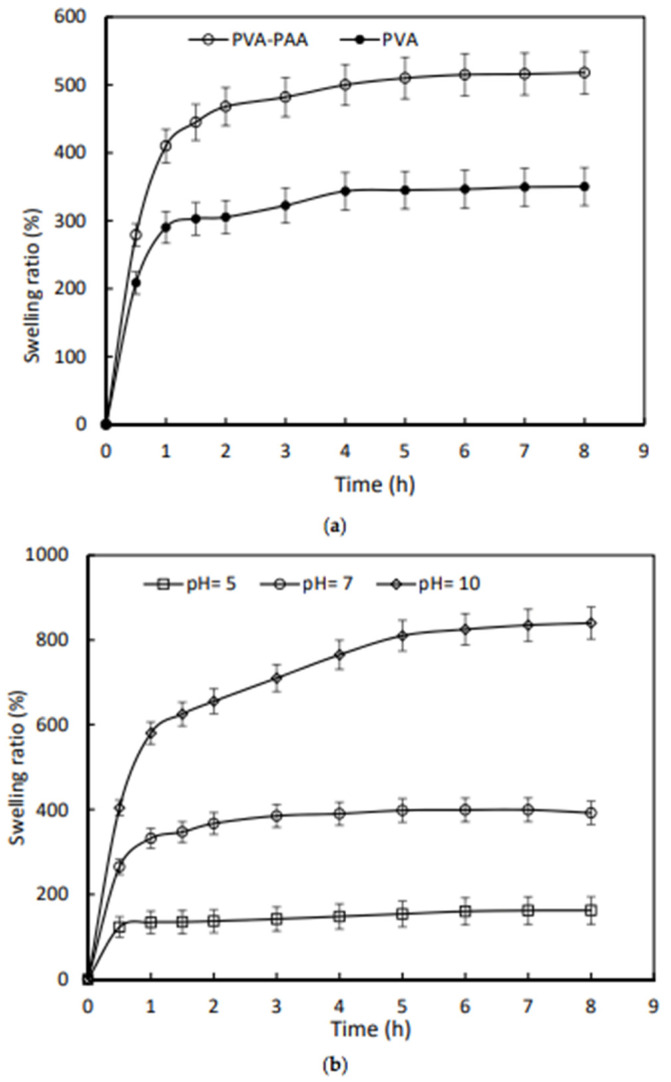
(**a**) The swelling properties of the PVA-PAA and PVA samples in distilled water; and (**b**) the kinetics of swelling for the PVA-PAA samples at different pH values [120].

In contrast, by selecting synthetic polymers as materials for hydrogels, it is possible to control the mechanical properties of the dressing. Leading positions are occupied by polyacrylic acid-polyvinyl alcohol (PAA-PVA) mixtures as components for biomedical applications dressings due their biocompatibility and biodegradability [121]. The encapsulation of polyacrylate layers into a bilayer hydrogel improves the performance of the resulting material such as moisture absorption, while minimizing the influence of interface-binding on the water uptake capacity of PVA [123]. The equilibrium swelling coefficient of the PVA-PAA hydrogel increased by 48%. The PVA-PAA hydrogel was also sensitive to pH changes; at various pH values of 5 and 10, the degree of equilibrium swelling changed by 115% and −58%, respectively, compared with the index at pH = 7. The equilibrium swelling time was measured as 7, 3, and 1 h for the PVA-PAA hydrogel at various pH values of 10, 7, and 5, respectively. Moreover, it was possible to control the drug release by the pH increase of infected wounds due to the fact that the release rate for tetracycline was found at a higher pH = 8 compared to neutral pH (Figure 9). 

**Figure 10:**
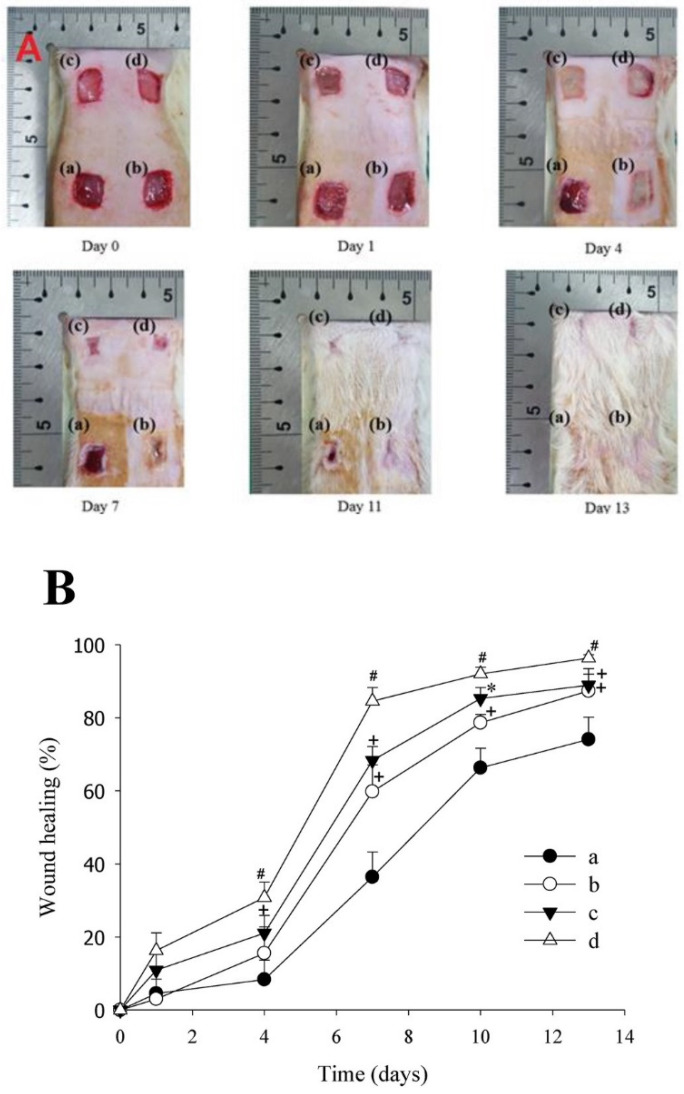
Representative images (**A**) and wound recovery profile (**B**) in a wound infected with *Pseudomonas aeruginosa*: (**a**) non-treated; (**b**) commercial product; (**c**) CDD without LB; (**d**) LB-loaded CDD. + *p* < 0.05 compared with non- treated, * *p* < 0.05 compared with non-treated and commercial product, # *p* < 0.05 compared with the non-treated, commercial product, and CDD without LB. CDD—composite double-layered dressing; LB—*Lactobacillus brevis*. Reproduced from [124], with permission from Elsevier, 2022.

An example of a sensitive component of a bilayer material is a thermosensitive drug of Lactobacillus brevis (LB) [124]. The bilayer hybrid material consists of a hydrogel layer filled with LB for the controlled release of the active compound, which has pro-angiogenic and antimicrobial activity for wound healing, and a hydrocolloid-type outer layer to improve the mechanical properties. The top layer of the hydrogel consisting of PVA and PVP was prepared by the freeze–thaw method. To compare the healing ability, the developed two-layer coating was compared with the effect of a commercial product (Duoderm^TM^) on rat wounds infected with *P. aeruginosa*, where the Lactobacillus brevis treated wound demonstrated faster healing (Figure 10). 

A hydrogel’s antibacterial resistance can be exhibited without drugs by the modification of polymers [125]. A bilayer hydrogel material consisting of N-isopropylacrylamide and a chitosan-N-2-hydroxypropyltrimethylammonium chloride (HACC) inner layer (Hm-PNn) and a polyvinyl alcohol and acrylamide outer layer (PVAo-PAmp) was covalently crosslinked by the photoinduced electron/energy transfer polymerization-reversible addition-fragmentation with chain transfer (PET-RAFT). The antibacterial resistance of the material was manifested by *Staphylococcus aureus* as a Gram-positive bacteria and *Escherichia coli* as a Gram-negative bacteria due to the components of the lower layer. At the same time, the outer layer, exposed to the environment, had good extensibility and strength. The results of the in vivo tests demonstrated the improved collagen location and granulation tissue thickness, which proved the effect of the Hm-PNn/PVAo-PAmp hydrogel material on the acceleration of wound healing, which was demonstrated in a full-thickness skin defect model demonstrating the improved collagen location and granulation tissue thickness.

**Figure 11:**
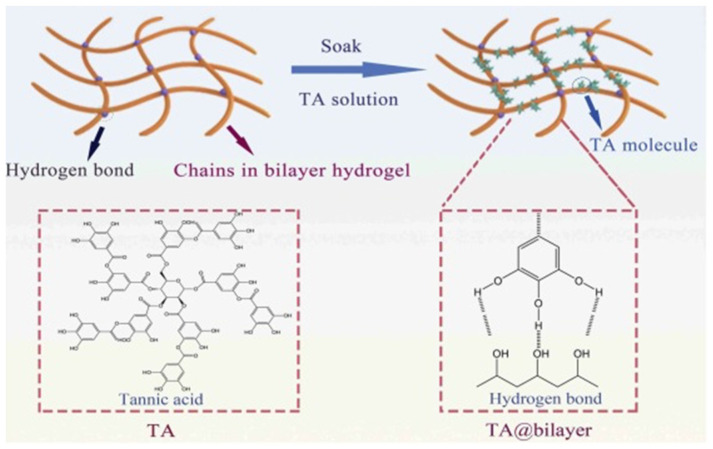
The crosslinking mechanism diagram of the TA@bilayer hydrogels. Reproduced from [126], with permission from the Royal Society of Chemistry, 2022.

Hydrogels with tannic acid have antibacterial and antioxidant properties, which in turn facilitates the process of recovery and healing. The accelerated wound healing when using a hydrogel dressing with a two-layer structure with different pore sizes to prevent bacteria from entering into the wound and control moisture content and gas exchange, was proven [126]. In addition, it is worth noting that the resulting hydrogel had shape memory and self-healing ability due to the hydrogen bonds that formed between TA and the bilayer hydrogel (Figure 11). Hydrogel dressings are designed to control fluid exchange on the wound surface, accelerate regeneration and recovery, and prevent infection from the entering into the body [127,128,129].

**Figure 12:**
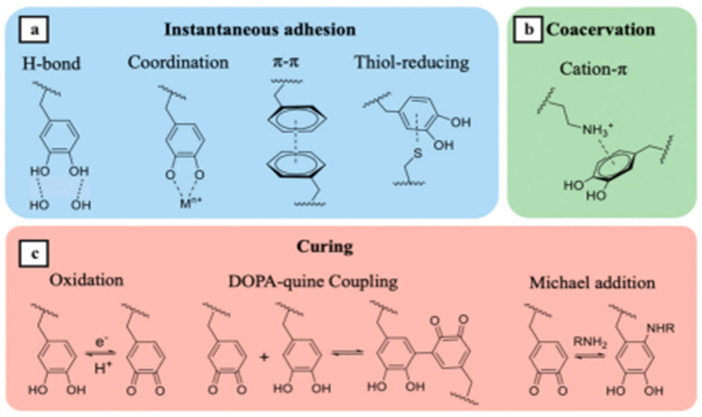
The multiple roles of catechol groups in wet adhesion: (**a**) instantaneous adhesion, (**b**) coacervation, and (**c**) wet adhesive curing. Reproduced from [131], with permission from Elsevier, 2022.

The reason for the adhesiveness of hydrogels is the continuous formation of catechin groups as a result of a dynamic redox system [130]. The Fe^3+^/TA-CN system triggers a dynamic redox system of catechin groups; the activated potassium persulfate initiator forms a large amount of free radicals for accelerated polymerization, even at low temperatures [131,132] (Figure 12). The author confirmed the preservation of the adhesiveness of the material during storage under extreme conditions for a long time. 

One of the most important processes in wound healing is the assessment of the wound state. In the treatment of skin diseases, bilayer hydrogels can act as a highly adhesive sensor in the form of an ultra-extensible wear-resistant sensor [133].

**Figure 13:**
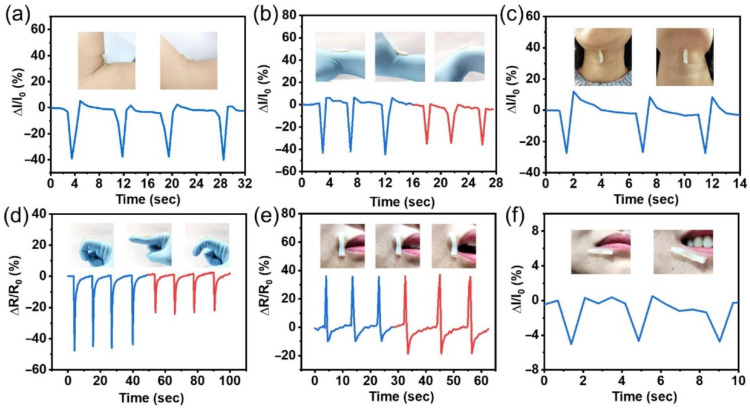
Human motion detection. (**a**) Electrical signal response of the bilayer composite hydrogel strain sensor with arm bending. (**b**) The relative current of the strain sensor changes with the wrist moving in two directions. (**c**) The piezoresistive response of the strain sensor when nodding. (**d**) The electrical signal of the strain sensor response with finger bending at 45° and 60°. (**e**) The relative resistance changes of the strain sensor when the mouth is opening. (**f**) Current response curves for smiling. Reproduced from [134], with permission from Elsevier, 2022.

The elastic upper layer, consisting mainly of synthetic polymers and integrated cellulose nanofibers with high extensibility and strength, plays the role of a sensor; and the lower layer, consisting of tannin and proline, has high biocompatibility, which accelerates wound healing and also increases the adhesion of the dressing to the skin [134]. In real-time, the hydrogel strain sensor has the ability to monitor the physical activity of various human movements such as the opening of the mouth, curling fingers, smiling, and diagnosing health status. The strain sensor can also detect both large-scale and small movements of the human body. During flexion movements of the fingers, the strain sensor demonstrated a clear discrepancy in the changes in the relative resistance when bending the finger with different amplitudes, which indicates the possibility of distinguishing the flexion movements of different fingers. When applying a load cell near the mouth, a significant change in the current during a smile was noticed, which lays the foundation for the accurate recognition of facial expressions (Figure 13). 

To characterize the sensitivity of a strain gauge sensor, a strain gauge coefficient (GF) is introduced, which is calculated by the formula:

(6) $$GF=\frac{\left(\Delta R/R_{0}\right)}{\varepsilon }$$

where *R*_0_ is the initial resistance; Δ*R* is the changes in resistance; and *ε* is the applied strain.

### 2.2. Bilayer Hydrogels as Scaffold for Tissue Engineering Applications

Hydrogels act as scaffolds for the delivery system of cells and biochemical factors in tissue engineering, having the form of a three-dimensional substrate that allows cells to attach, proliferate, and grow, resulting in the regeneration of new tissue [135,136].

Cell therapy promotes additional therapeutic possibilities that contribute to faster wound healing and restoration of normal skin architecture.

Scaffolds made from natural polymers are biocompatible, biologically active, promote cell attachment to cell surface receptors, and provide a niche for cell function control [137]. One example of such biopolymer is collagen. Attempts have been made to mimic the natural bilayer organization of the skin using porous scaffolds based on collagen (Coll) for the epidermis and sodium carboxymethyl cellulose (NaCMC) for the dermal layer [138].

One of the main parameters of the hydrogel scaffold is the pore size. One example of a study of size variation for wound treatment was the biomatrix of a bilayer gelatin-CS-hyaluronic acid hydrogel [139]. Chondroitin-6-sulfate and hyaluronic acid were included in the gelatin matrix to mimic the composition of the skin and to create a suitable microenvironment for cell proliferation, differentiation, and migration. The bottom layer was seeded with dermal fibroblasts and served as a feed layer for keratinocyte inoculation (pore size: 150 µm) while the upper layer was seeded with keratinocytes for epidermalization (pore size: 20–50 μm)**.**

The top layer seeded with keratinocytes developed into an epidermis-like structure, and the bottom layer seeded with dermal fibroblasts developed into a dermis-like structure after culturing the cells for 21 days. Histological studies and immunostaining have proven that keratinocytes form multi-layered layers of the epidermis within 21 days. The results showed that, in addition to the permanent coverage of histologically healthy and differentiated epithelial tissue, there was a well-defined dermo-epidermal junction and collagen network in the dermis. Importantly, pore size plays an important role in cell migration within the membrane. The micropores of the upper layer (pore size: 20–50 µm) not only allow for the migration of keratinocyte populations from the upper surface into the wound, but also serve as a substrate to prevent excessive spread of keratinocytes into the dermal layer, while seeded on the macroporous (pore size: 150 µm) on the top layer of the membrane part, dermal fibroblasts can act as a feeding layer for keratinocytes, and the porous structure of the hydrogel is used to remove the wound exudate.

**Figure 14:**
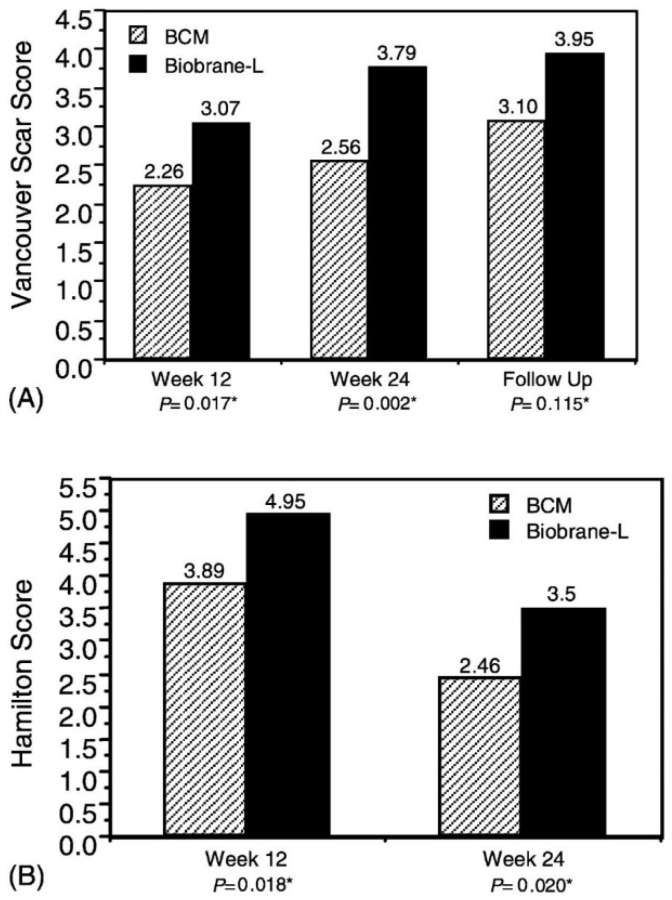
Scarring severity. At weeks 12 and 24, wounds were assessed for scarring using the Vancouver Scar Scale (**A**) and the Hamilton Burn-Scar Rating Score (**B**). Scar severity at the follow-up visit was also determined using the Vancouver Scar Scale (**A**). The data represent the mean total scarring severity. BCM indicates a bilayered cellular matrix (OrCelTM). * Paired *t*-test. Reproduced from [140], with permission from Elsevier, 2022.

Hydrogel combinations with materials of a different nature that exist in the form of foams, sponges, and nanofibers can serve as an alternative to bilayer materials based on hydrogels for skin regeneration. Most porous scaffolds seeded with keratinocytes or fibroblasts are based on collagen [140]. The two-layer sponge structure has a top layer of non-porous collagen gel, on top of which keratinocytes are added, and a layer below it is a porous collagen sponge, where fibroblasts are seeded. In a non-toxicity and efficacy study, a bilayer cell matrix (OrCel™) from Ortec International, Inc. (New York, NY, USA) to facilitate the timely wound closure of varying thicknesses of donor skin in patients with severe burns was compared with a commercial single layer analogue Biobrane-L^®^ (Bertek Pharmaceuticals, Sugarland, TX, USA). Donor sites treated with OrCel^TM^ were examined for the severity of scarring. Investigators undertook assessment at weeks 12 and 24, and at subsequent visits twice a year using the Vancouver Scar Scale, which assesses pigmentation, vascularity, compliance, and height of the donor site, and the Hamilton Burn-Scar Rating Scale, which was conducted by blind viewing (Figure 14). 

The results showed that OrCel™ was more effective: it was recorded that the healing rate for the treated areas of OrCel™ was significantly faster.

Hybrids of a bilayer structure to accelerate wound healing can be made from keratin and chitosan nanofibers with a gelatin methylacrylate hydrogel layer [141]. The synthesis technique makes it possible to obtain a two-layer multifunctional structure with a high water content to mimic tissue with human keratinocytes (hKC) and dermal fibroblasts (hDFb). To prepare the top layer of nanofibers, consisting of the solution of human hair keratin and chitosan (mixture ratio: 5/5), an electrospinning method was used with formic acid as a solvent in the presence of polyethylene glycol, followed by crosslinking with glutaraldehyde. The lower hydrogel layer was obtained by radical crosslinking of the solubilized gelatin derivatives. A bilayer structure was prepared by the photopolymerization of GelMA under a mat of crosslinked human hair keratin and chitosan nanofibers.

**Figure 15:**
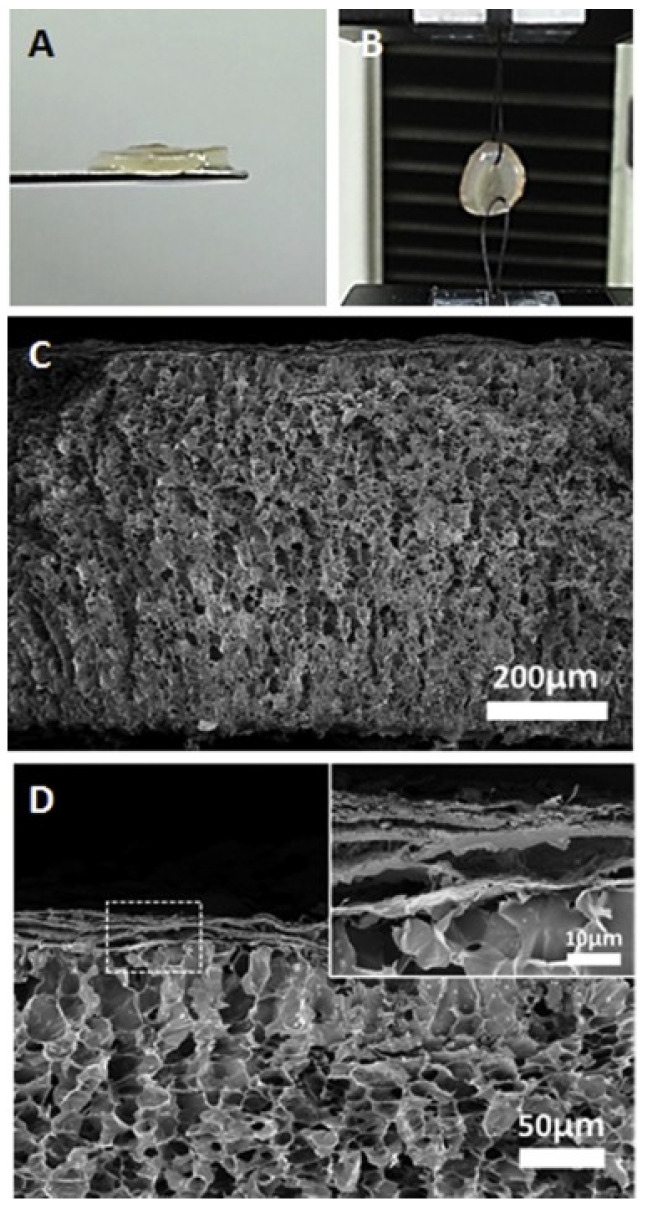
Photographs of the nanofiber-hydrogel bilayer scaffold: (**A**) side-view, and (**B**) sutured bilayer scaffold. (**C**,**D**) Cross-sectional FE-SEM images of the nanofiber-hydrogel bilayer scaffold. Reproduced from [141], with permission from Elsevier, 2022.

The GelMA hydrogel layer contained a huge amount of micropores with a diameter of 10–20 µm, while the shear modulus was about 0.5 kPa. The 30 µm thick nanofiber layer was firmly bonded to the bottom hydrogel layer without any separation (Figure 15). 

The inclusion of chitosan in the nanofibers improved the mechanical stability of the material. To assess the effectiveness of the scaffold, human fibroblasts were placed in the hydrogel layer and HaCaT cells were cultured on the layer of nanofibers and co-cultured for 10 days. As the result, encapsulated fibroblasts proliferated in the hydrogel matrix, and HaCaT cells formed a cell layer on top of the scaffold, imitating the dermis and epidermis of skin tissue.

**Figure 16:**
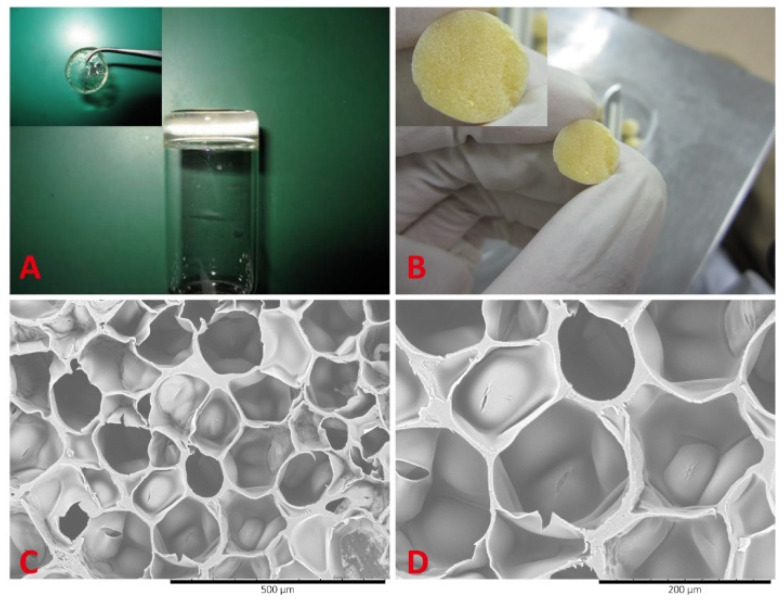
The morphology of the dextran/gelatin hydrogels. (**A**) Gross view of the dextran/gelatin hydrogel. (**B**) The gross view of the lyophilized dextran/gelatin hydrogel. (**C**) The SEM micrograph of the lyophilized dextran/gelatin hydrogel. Scale bar represents 500 mm. (**D**) The SEM micrograph of lyophilized dextran/gelatin hydrogel. Scale bar represents 200 mm [142]. Reproduced from [142], with permission from Public Library of Science, 2022.

The composition based on poly(ε-caprolactone-colactide)/poloxamer (PLCL/poloxamer) nanofibers as the upper layer and a hydrogel consisting of dextran and gelatin as the lower layer formed a material with increased mechanical stability and supported cell proliferation [142] (Figure 16). 

The upper layer of the nanofibers demonstrated high biocompatibility and mechanical protection of the material, while the lower hydrogel dextran/gelatin (5/5) was a rapidly forming scaffold in situ, combining the ability to maintain cell viability and the mechanical strength necessary for skin scaffolds. The pore sizes of the hydrogel layer were about 50 to 200 microns. It is important to note that the porous structure of the scaffolds supports the diffusion of nutrients and gases, allows for growth cell, and maintains a high water level. Dry hydrogels can absorb large amounts of water from 19.47 to 43.45 g/g. At the same time, the degree of swelling changes nonmonotonically with the increase in the concentration of dextran in the hydrogel. The degree of swelling decreases rapidly as the dextran content increases from 30 to 50%, which can be explained by the increase in the crosslink density. With an increase in the dextran content from 50 to 70%, the crosslink density in hydrogels decreased, and the degree of swelling slightly increased from 19.47 to 25.94 g/g. In addition, the concentration of dextran and, accordingly, the density of crosslinking, played important roles in the controlled degradation of the material over a certain period of time, which, in turn, creates a comfortable environment for the formation of epidermal tissue. The mechanical properties of dextran/gelatin hydrogels are highly influenced by the mass ratio of monomers, which may be associated with pore size and water content.

The study results under a fluorescent microscope revealed no difference in the effect on the vital activity of the cells of the control group. The group with a bilayer PLCL/poloxamer nanofibers and dextran/gelatin hydrogels scaffold was not found, thereby confirming the biocompatibility of the degradation products.

Gelatin-based hydrogels are scaffolds for skin regeneration, with the inclusion of keratinocytes and fibroblasts [143]. The two-layer skin substitute, “PG-1” or “first generation pullulan-gelatin hydrogel”, with the addition of human fibroblasts and keratinocytes in vitro, is characterized by low cost due to the use of pullulan—a polysaccharide with antioxidant properties and gelatin with a high sorption capacity. The investigation into the material demonstrated the optimal mechanical characteristics for a hydrogel as a skin substitute such as an average pore size of 61.69 μm with an ideal elastic modulus, swelling behavior, and biodegradability. Compared to the cell-free PG-1 study in vivo, which showed that cell encapsulation increased proliferation, the thickness of the neodermis layer was 204.00 ± 19.65 μm compared to acellular hydrogels of 115.63 ± 9.83 μm, and the controls, after the skin biopsy. Furthermore, PG-1 provided cell viability and angiogenesis and led to inflammation reduction and macrophage infiltration.

Crystallization is one example of the physical crosslinking of gels, involving a freeze/thaw process [144]. A two-layer wound dressing made of polyvinyl alcohol/carboxymethyl cellulose/polyethylene glycol (PVA/CMC/PEG) with a gradual change in pore size was developed using freeze–thaw and phase separation methods [145]. The study results showed increasing pore sizes from the upper to the low layer. The pore sizes were 20 μm and 100 μm in the upper dense layer of the wound dressing and the lower porous layer, respectively. 

**Figure 17:**
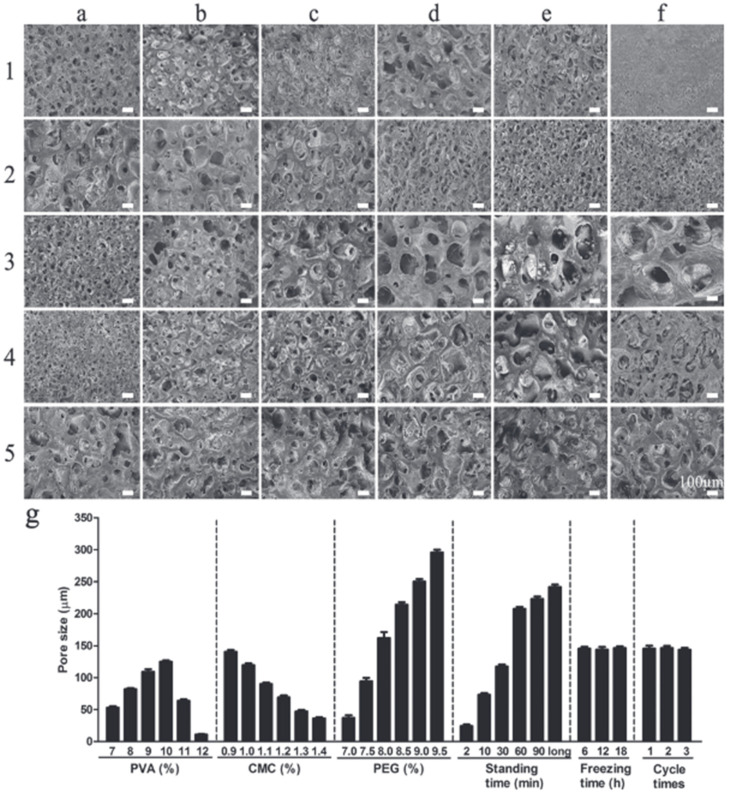
A study of the pore sizes of a single-layer PVA/CMC/PEG hydrogel. The first line represents the hydrogels with different concentrations of PVA, from (**a1**–**f1**), the concentrations are 7%, 8%, 9%, 10%, 11%m and 12%, respectively. The second line refers to the hydrogels with various concentrations of CMC, from (**a2**–**f2**), the concentrations are 0.9%, 1.0%, 1.1%, 1.2%, 1.3%, and 1.4% in sequence. The third line denotes the hydrogels prepared with different concentrations of PEG, and the concentrations are 7.0%, 7.5%, 8.0%, 8.5%, 9.0%, and 9.5% from (**a3**–**f3**). The fourth line represents hydrogels prepared on the condition of different standing times at room temperature, from (**a4**–**e4**), the standing times are 2, 10, 30, 60, and 90 min; (**f4**) refers to the hydrogel gelled at room temperature but −20 °C. The last line represents the hydrogels prepared for different freezing times and freezing–thawing cycle times; (**a5**–**c5**) represent freezing times of 6, 12, and 18 h, and (**d5**–**f5**) represent freezing–thawing cycle times of 1, 2, and 3. The scale bar is 100 μm. (**g**) shows the relationship between the content of the components and pore size. Reproduced from [145], with permission from John Wiley and Sons, 2022.

The freeze–thaw cycles did not affect the pore size, which confirmed the pore formation during the phase separation at room temperature, while as the exposure time increased, the pore sizes constantly increased. In addition, the concentration of polymers affected the pore size. As the PVA concentration increased (from 7% to 10%), the pore sizes first increased and then decreased. This phenomenon can be explained by the increase in the viscosity of the solution, which prevents the phase separation due to the decrease in fluidity. The increase in PEG concentration from 7.0% to 9.5% led to the increase in pore size, where PEG plays the role of a pore former. At a low PEG content, the degree of phase separation was weak. The increase in the CMC concentration led to a decrease in pore size, which the authors suggest is due to the nature of CMC, as it is a surfactant that can reduce the surface tension, thus making the dissolution of PEG easier and more uniform. The increase in the CMC concentration increases the viscosity of the system, which slows down the phase separation, while insufficient viscosity, on the other hand, cannot lead to the phase separation (Figure 17).

**Figure 18:**
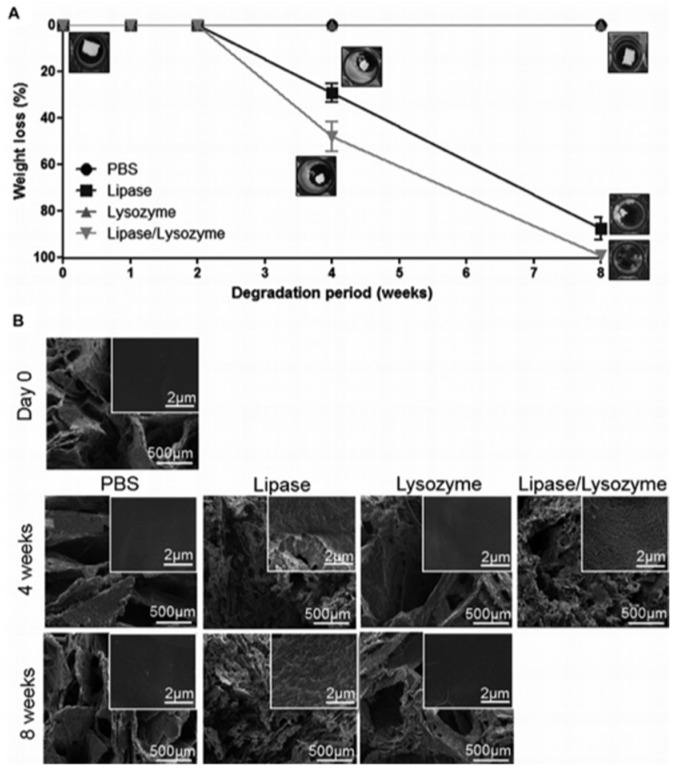
The degradation of PHBV freeze-dried scaffolds. (**A**) Percentage of weight loss along the immersion time in PBS, lipase, lysozyme, and lipase/lysozyme, at pH 7.4 and 37.8 °C. The macroscopic images represent the appearance of the freeze-dried scaffolds under the different conditions at specific time points. (**B**) The SEM images of the PHBV scaffolds before (day 0) and after 4 and 8 weeks of immersion in PBS, lipase, lysozyme, and lipase/Lysozyme. Images in the upper right corners represent magnified areas highlighting the topography of the pore wall surfaces. Samples immersed in lipase/lysozyme solution for 8 weeks were completely degraded and therefore no image corresponding to that condition is shown. Reproduced from [146], with permission from John Wiley and Sons, 2022.

Zonari et al. designed a polyhydroxybutyrate-co-hydroxyvalerate (PHBV) bilayer skin tissue scaffold considering the two main layers of the skin: the epidermis and dermis. The new material was a thin membrane and a highly porous structure that was constructed by the combination of both types of methodologies, created through membrane solvent casting and the 3D freeze-dried scaffold, respectively [146]. The skin scaffold demonstrated high water retention capability and controlled stability against degradation by enzymes. One of the main disadvantages of PHBV is the hydrolytic degradation [147,148,149]. Degradation studies revealed the resistance to the action of lysozyme within 8 weeks of the treatment, the action of lipase led to the change in the weight of the material by almost 90% over the same period, while the co-treatment of the two enzymes led to the complete destruction of the material (Figure 18). Enzyme concentrations were approximately equal to the blood serum concentrations.

Kamali et al. presented the development of a bilayer skin scaffold consisting of a layer obtained by the electropressing of polycaprolactone and PVA, and a porous hydrogel layer made of chitosan and gelatin. The hydrogel layer was obtained using a lyophilization method to obtain a porous structure in the hydrogel [150]. The authors compared the characteristics of the two-layer structure with a single-layer chitosan-gelatin hydrogel. The mechanical strength and modulus tests of the combination of the hydrogel layers and electrospinning resulted in strength increases of 110% and 133% compared to the single layer hydrogel sample (Table 6).

**Figure 19:**
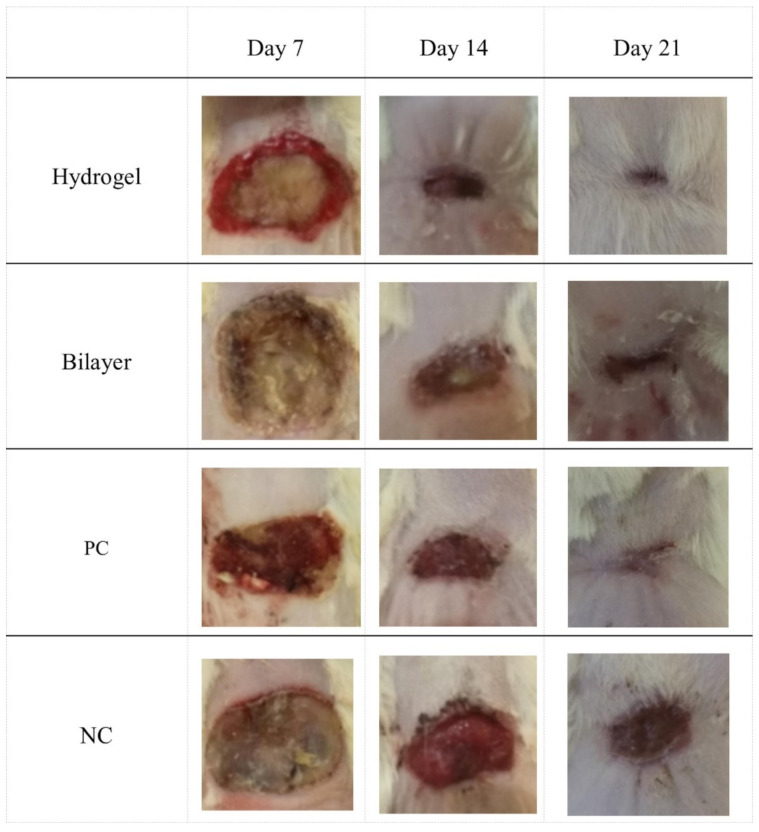
Wound images on inspection days (days 7, 14, and 21). Reproduced from [150], with permission from Elsevier, 2022.

It is important to note that the gelation step does not damage the electrospun layer of the bilayer structure during the manufacturing process. At the same time, significant superiority of the two-layer structure was not observed in the study of sorption capacity. The study results of the histopathology and histomorphometry showed that the wounds treated with a bilayer scaffold showed the fastest healing: epidermal proliferation and an increase in the thickness of the epidermal layer were observed 21 days after the treatment. A decrease in the inflammatory response and granulation tissue was also observed (Figure 19).

Keratinocytes were co-cultivated with fibroblasts in the chitosan–gelatin–hyaluronic acid scaffold to construct an artificial bilayer [151]. The study results of the physicochemical properties suggested that the porosity and pore size of the scaffolds could be modulated by the thermodynamic and kinetic parameters of the freeze-thawing process. The encapsulation of hyaluronic acid led to the increase in the sorption capacity of the material and to a long-term retention of water compared to the single-layer hydrogels. Fibroblasts cultured in chitosan–gelatin–hyaluronic acid scaffolds grew and proliferated well and showed high viability. Keratinocytes were co-cultivated with fibroblasts in chitosan–gelatin–hyaluronic acid scaffolds to create artificial bilayer skin in vitro.

A scaffold made of polycaprolactone electrospinning membrane and poly(lacto-glycolic acid) (PCL/PLGA) and cross-linked with glutaraldehyde (3.5% by volume) with a chitosan/gelatin hydrogel was fabricated using two methods: electrospinning of the membrane onto the lyophilized hydrogel (BS-1) and the membrane underlaying and casting method (BS-2) [152,153]. However, glutaraldehyde negatively affected cell proliferation [154]. The bilayer scaffold consisted of galvanized polycaprolactone/poly(lacto-glycolic acid) (PCL/PLGA) and chitosan/gelatin hydrogel crosslinked with glutaraldehyde. The cytotoxicity of the bilayer scaffold and chitosan/gelatin hydrogel (CGH) was compared. The thickness of the hydrogel was 2.13 ± 0.22 mm at a density of 0.125 ± 0.002 g/cm^3^, its porosity was 97.49%, and the average pore diameter was 290 ± 109 mm. The difference in the membrane layer was noted. The membrane deposition in BS-1 had a rough, variable microsurface while the membrane adhesion in BS-2 resulted in a flat and smooth appearance. 

A membrane layer was added to maintain the mechanical stability of the scaffold during the implantation and to withstand suturing. However, it was noted that the hydrogel layer degraded much faster in vivo compared to the in vitro experiments. Thus, the authors of the study suggest that this material can be used as a temporary skin substitute or tissue bandage.

The results showed that while the PCL/PLGA membrane supported fibroblast cells, the decrease in cell viability was observed in the hydrogel–gelatin–chitosan–glutaraldehyde due to the remaining glutaraldehyde content. These results show that the addition of a membrane layer to the hydrogel reduces the rate of swelling and degradation, which in turn opens up prospects for implantation.

The method of creating artificial skin using 3D bioprinting has become an innovative method in the area of 3D printing development [155]. A bilayer structure consisting of dermal fibroblasts, keratinocytes, and microvascular endothelial cells was designed and fabricated using an extrusion 3D printer to create micro vessels in skin grafts. Human dermal fibroblasts and microvascular endothelial cells were mixed with a gelatin–alginate composite hydrogel as the dermis, and human keratinocytes were mixed with the gel as the epithelium.

**Figure 20:**
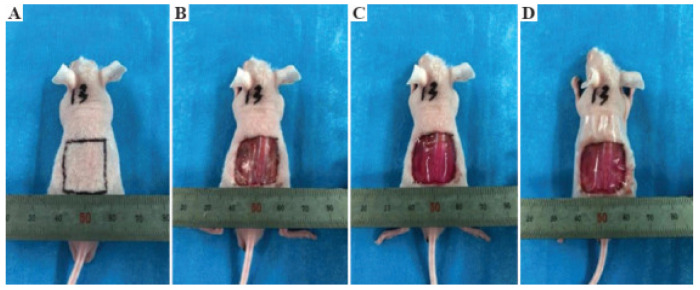
The gross observation of the mice after 4 weeks. (**A**) The printed skin graft, (**B**) control group without endothelial cells, (**C**) control group with no cells, and (**D**) the blank group. Reproduced from [155], with permission from Whioce Publishing Pte. Ltd. (Singapore), 2022.

The material obtained by 3D printing increased cell survival by more than 90%. The results of the histological and immunohistochemical analyses showed that two-layer constructions contributed to the healing and contraction of skin wounds with the predominant regeneration of micro vessels (Figure 20).

With the development of 3D skin printing technology, a bilayer membrane (BLM) scaffold was designed and printed consisting of a poly(lactic-glycolic acid) (PLGA) outer membrane and an alginate hydrogel bottom layer that respectively mimicked the epidermis and dermis of the skin [156]. The multi-porous alginate hydrogel of the BLM scaffolds promoted cell adhesion and proliferation in vitro, while the PLGA prevented bacterial invasion and the membrane maintained the moisture content of the hydrogel.

A double-layer membrane showed excellent ability in promoting neovascularization and collagen I/III deposition after implantation in the wounds of dorsal rats, which ultimately accelerated the wound healing process compared to the PLGA and alginate hydrogel control groups.

The 3D printing possibilities have served in the fight against diabetic wounds, characterized by long-term chronic inflammation, and reduced granulation tissue formation and vascularization. A bio-layer skin substrate was created consisting of an upper layer made of a gelatin cryogel with silver and a lower layer of platelet-derived growth factor-BB (PDGF-BB) with a 3D printed gelatin scaffold [157]. The bilayer scaffold application led to a high level of skin regeneration due to the increased neovascularization and collagen I/III deposition. The 3D printed BLM scaffolds promoted wound healing and so are suitable for a wide range of applications as wound dressings or skin prostheses. The substrate could not influence the proliferation of fibroblasts, keratinocytes, U937 cells, and HL60 cells. The CFU quantification results demonstrated that the scaffold showed good antibacterial ability, namely, the release of silver nanoparticles was able to significantly kill *Pseudomonas aeruginosa*, *Staphylococcus aureus,* and *E. coli*. Moreover, the substrate was able to promote re-epithelialization, granulation tissue formation, collagen deposition, and angiogenesis in vivo. The PDGF-BB-loaded scaffolds, silver and PDGF-BB coloaded scaffolds were able to accelerate wound closure, re-epithelialization, granulation tissue formation, and angiogenesis compared to the scaffold and the silver-loaded scaffold groups in vivo at each indicated time point.

Problems of skin scarring and reduced wound contraction with accelerated wound healing were demonstrated using a gelatin/sodium alginate/gelatin methacrylate bioprinter supplemented with normal human dermal fibroblasts (NHDF) and normal human epidermal keratinocytes (NHEK) to print the dermis and epidermis. The results showed accelerated epithelialization as well as the absence of scarring [158]. The multilayer hydrogel material gelatin/SA/GelMA had a high elongation at break, so the authors of the study suggest that the risks of tearing caused by the wound activity in the grafted skin are minimal (Table 7).

Stem cells are formed at the stage of embryonic development and their main distinguishing ability is their differentiation into specialized cells that can participate in the formation of skin prostheses. The main sources of cells that can be used to model such prostheses are adult stem cells, embryonic stem cells (ESCs), and induced pluripotent stem cells (iPSCs) [159].

It has been shown that stem cell therapy has the potential to treat chronic wounds, but has not been very successful in clinical practice. Research is currently underway to create suitable polymeric stem cell matrices to promote paracrine activity, which ultimately affects the healing process.

**Figure 21:**
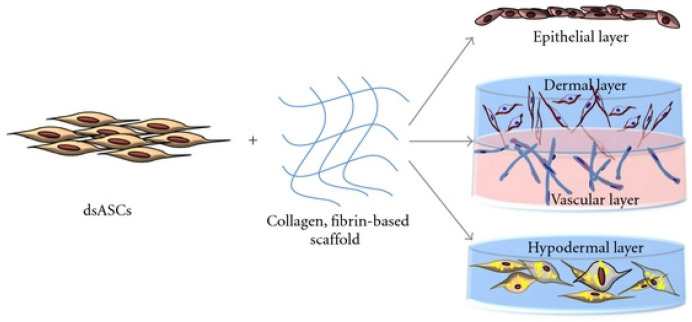
The development of different layers of skin substitute using dsASCs and hydrogel-based matrices. The epithelial and hypodermal constructs were developed using a collagen hydrogel and the vascularized dermal construct using a collagen-PEGylated-fibrin-based bilayer hydrogel. Reproduced from [162], with permission from Hindawi Limited, 2022.

Natesan et al. developed a bilayer hydrogel based on collagen and pegylated fibrin to deliver adipose-derived mesenchymal stem cells (ADSCs) derived from burnt skin layers. This work demonstrated the successful differentiation and proliferation of ADSCs, which formed dense tubular microvascular networks based on PEGylated fibrin. The bilayered hydrogels’ influence on wound healing was demonstrated by considering the wound process of rats with a control group. Wounds treated with bilayer hydrogels showed less wound shrinkage and better dermal matrix deposition and margin progression. In addition, in the experimental rats, the significant increase in the formation of granulation tissue and re-epithelialization of the wound edges were observed [160,161]. Similar cells in a bilayer hydrogel based on polyethylene glycol and fibrin differentiated into an epithelial layer, a vascularized dermal layer, and a subcutaneous layer. Polytransretinoic acid and fenofibrate have also been used to differentiate dsASCs into epithelial-like cells (Figure 21) [162].

## 3. Bilayer Gradient Hydrogel Materials

Due to their properties, namely, the ability to retain a large amount of water, mimic tissue, and act as a cellular microenvironment, hydrogels are widely used in tissue engineering. However, the absolute identity of the cellular environment and the extracellular matrix is impossible due to differences in the physical and chemical properties [163]. Functional gradient hydrogels provide an optimal heterogeneous cellular environment. The encapsulation of physical and chemical gradients into three-dimensional scaffolds solves one of the main problems of tissue engineering—the detection of the cellular and tissue environment in vivo [164].

In a broad sense, a “gradient hydrogel” is defined as a macromolecular structure with a space–time change in at least one of their physico-chemical characteristics (that is, those that gradually change over a certain period in space and may even evolve over time) [165]. Physical gradients regulate cellular behavior: motility, migration, signaling, differentiation, and proliferation. Physical gradients are divided into classes: stiffness gradients and pore size/porosity gradients. Chemical gradients are defined as morphogens—bioactive substances such as transcription factors, chemokines, and cytokines that give direction to cell growth. Morphogens are signaling molecules that can induce different cellular responses depending on the concentration. The spatial distribution of proteins provides biochemical signals for organized tissue formation [166]. Chemical gradients can be divided into two categories: immobilized gradients, where the molecules are tightly bound to the extracellular matrix, and diffusion soluble factor gradients, which are loosely bound [167]. At the moment, in order to study the cellular response, growth factors and peptides in absolute and relative concentrations are immobilized in skin scaffolds [168]. 

Methods for the fabrication of gradient hydrogels including photolithography [169], bioprinting [170], diffusion systems [171], and microfluidics [172] have been developed. Zang et al. developed a biomaterial based on collagen (Col), hyaluronic acid (HA), and chitosan (Chs) with tyrosinase as an addition for a three-dimensional cell structure with a high degree of biodegradation and biocompatibility [173]. The uniqueness and prospects of this work lie in the combination of crosslinking methods: preliminary chemical crosslinking before the printing and physical crosslinking after. The study results showed that the scaffolds demonstrated improved mechanical properties and biocompatibility with the help of a modernized crosslinking process. In addition, through the presence of the concentration of tyrosinase as crosslinking agent, it was possible to control the mechanical strength and degradation rate. Enzymatic crosslinking is an attractive technique as it allows for desirable properties of the resulting material such as controlled in situ gelation by controlling the enzyme concentration and strong covalent bonding [174]. The sample without an enzyme content was used as the control group. The tensile strengths of Chs/HA/Col and Chs/HA/Col-T were 29.9 and 39.1 kPa, respectively. The Young’s modulus of Chs/HA/Col and Chs/HA/Col-T was 0.170 and 0.313 MPa, respectively, which provided the increased mechanical strength, which corresponds to human skin. The Chs/HA/Col and Chs/HA/Col-T degradation rate at 35 days was 81.99% and 55.34%, respectively.

**Figure 22:**
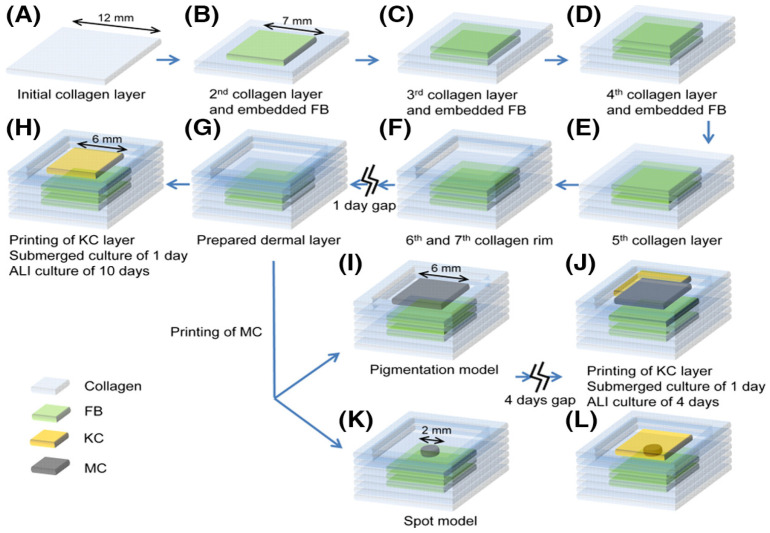
The schematics of printing biomimetic skin constructs including the ALI culture periods. (**A**–**G**) Method of printing the FB-containing dermal construct and (**H**) the KC layer. The KC/FB composites were cultured in the submerged condition for 1 day, and then in the ALI condition for 10 days. The dermal layer underwent the pigmentation procedure by (**I**) printing the MC in the square area (6 mm side), or (**K**) in a single spot (2 mm in diameter). After 4 days of culturing the MC-laden dermal layer, (**J**,**L**) the KC were printed on top to make the epidermal layer. The KC/MC/FB constructs were cultured in the submerged condition for 1 day, and then in the ALI condition for 4 days. The overall time for the culture period was similar for both skin constructs, without or with MC. Reproduced from [175], with permission from John Wiley and Sons, 2022.

Min et al. created a full-size skin model with pigmentation. Initially, many collagen-based hydrogel layers containing fibroblasts that mimicked the skin layer were “printed” (Figure 22). Then, melanocytes and keratinocytes were printed on top of the layer to stimulate pigmentation. The bioprinted skin scaffold imitated the skin regeneration, epidermal, and corneal layers [175]. There were also freckle-like pigmentations at the dermal-epidermal junction shown in the MC-containing epidermal layer. 

## 4. Conclusions and Future Prospects

Hydrogels are promising types of materials that have already entered into the commercial market with successful performance indicators. However, the various hydrogel wound dressings presented on the commercial market do not meet all of the requirements, and along with the quality, have a number of disadvantages—toxicity, allergenicity, mechanical instability, etc. In this regard, there is a need to create and develop stable and multifunctional systems for the treatment of wounds of various natures. 

Hydrogel dressings are a promising direction in the treatment and care of wound surfaces as they meet all of the requirements of an ideal dressing: antimicrobial properties, adhesion and hemostasis, anti-inflammatory and antioxidant effects, drug delivery, self-healing, stimulus response, and conduction. Bilayer hydrogel dressings, due to their structure and variety of preparation methods, have the ability to combine the above-mentioned properties, which makes it possible to find their wide application in the field of biomedicine. It may be noted that the dressings developed currently have antibacterial and antioxidant properties that combine the effect of accelerating healing, the main components of which are commercial antibiotics and drugs, nanoparticles, and even modified polymers. It should be added that in the conducted studies, the influence of temperature and pH of the medium on the release was also investigated. In addition to antibacterial and wound healing properties, bilayer wound dressings have the ability to have a synergistic effect with the function of monitoring wounds.

Hydrogels are widely used in tissue engineering due to their ability to retain large amounts of water, mimic tissue, and act as a cellular microenvironment. Advances in stem cell biology and biomaterials have created exciting possibilities for wound healing. Polymeric biomaterials can now be engineered to act as the matrix needed for stem cell survival and function. Stem cell therapy has the potential to treat not only acute but also chronic wounds. In addition, the two layers, showing multifunctional properties, differentiate into different tasks: the top layer protects against external factors and maintains a humid environment, while the bottom layer promotes cell adhesion and proliferation.

However, the absolute identity of the cellular environment and the extracellular matrix is impossible due to differences in the physical and chemical properties. Bilayer functional gradient hydrogels provide an optimal heterogeneous cellular environment. Moreover, the ability to print gradient hydrogels allows for melanocytes and keratinocytes to be applied to the top layer for pigmentation stimulation.

Techniques for the creation of bilayer hydrogels are very diverse and inventive in their selection. The method of creating artificial skin using 3D bioprinting has become more attractive, allowing one to obtain a coating with an ordered structure, promoting complete skin regeneration with mechanical stability, reduce inflammation, and that is also applicable to diabetic wounds.

As an alternative to bilayer materials based on hydrogels for skin regeneration, their combinations with materials of a different nature, which exist in the form of foams, sponges, and nanofibers, are of interest for further developments.

Despite the intensive research and developments in this field, there is an almost complete absence of bilayer wound dressings on the commercial market due to the necessity of further pre-clinical and safety experiments.

## Figures and Tables

**Table 1:** Synthetic polymer systems for wound dressing.

Polymer Basis	Hydrogel Composition	Key Effects	Ref.
**PEG**	CSG-PEG/DMA6/Zn	Antibacterial properties against MRSA, compressibility, adhesion, antioxidation and hemostasis	[29]
	1. PBP—PEG 2. PDP—PEG	Injectable; antibacterial, anti-inflammatory effects	[30]
	HA-PEGSB-CMP	Injectable wound dressing, stretchable; self-healing; anti-oxidant promoting healing of infected by MRSA wound	[31]
**PAA**	PAA-HA-NHS	Elastic and adhesive properties	[32]
	PAA-PEA	Antioxidant property, hemostatic effects, accelerating healing	[33]
	Net(Agar/AAc)—AgNPs	excellent mechanical properties; antimicrobial activity against *E. coli*	[34]
**PU**	HPUC—PLGA	Antimicrobial wound	[35]

PEG—polyethylene glycol, CSG-PEG—polyethylene glycol monomethyl ether modified glycidyl methacrylate functionalized chitosan, DMA—methacrylamide dopamine, PBP—poly(N-butylimidazolium propiolic acid sodium), PDP—poly(N-(3,6-dioxaoctane) imidazolium propiolicacid sodium), HA—hyaluronic acid, PEGSB—poly(ethylene glycol)–co-poly(glycerol sebacate), CMP—cuttlefish melanin nanoparticles, PAA—polyacrylic acid, PAA-HA-NHS—poly(acrylic acid)—N-hydroxysuccinimide grafted hyaluronic acid, PAA-PEA—poly(acrylic acid) and antioxidant poly(ester amide, AAc—acrylic acid, PU—polyurethane, HPUC—polyurethane–chitosan hydrogel membrane, PLGA—poly(lactic-co-glycolic acid), MRSA—methicillin resistant *Staphylococcus* *aureus*.

**Table 3:** The release parameters of the ML-D hydrogels. Reproduced from [108], with permission from Elsevier, 2022.

Zero-Order Model		First-Order Model		Higuchi Model			Korsmeyer–Peppas Model	
k_0_	R^2^	k_1_	R^2^	k_H_	R^2^	k_KP_	*n*	R^2^
0.023	0.96	0.0039	0.88	0.58	0.90	0.035	0.41	0.91

**Table 4:** The bacterial reduction percent of the samples. Reproduced from [115], with permission from the Royal Society of Chemistry, 2022.

Sample	*E. coli* (%)	*S. aureus* (%)
SA	5.57 ± 4.02	11.97 ± 2.53
SAZn1	23.75 ± 1.77	23.95 ± 1.16
SAZn2	32.55 ± 2.11	33.65 ± 3.21
SAZn3	67.74 ± 0.76	68.38 ± 0.65

**Table 5:** Analyses of the release kinetics of CM from CMx-loaded SA hydrogels based on a Fickian diffusion type of release mechanism (*n* = 3) Reproduced from [122], with permission from Elsevier, 2022.

CaCl_2_ (% *w*/*v*)	Rate Parameter, *k* (s^−0.5^)	r^2^
0.05	0.0025	0.99
0.10	0.0022	0.77
0.20	0.0073	0.98

**Table 6:** The mechanical properties of the two comprising layers along with those of the bilayer scaffold. Reproduced from [150], with permission from Elsevier, 2022.

	Tensile Strength (kPa)	Elastic Modulus (kPa)
Electrospun sheet	5322.5 ± 345.1	35,080.6 ± 4121.3
Hydrogel	300.6 ± 27.4	438.4 ± 25.4
Bilayer	632.5 ± 54.2	1023.4 ± 72.5

**Table 7:** The mechanical properties of the gelatin/SA/GelMA hydrogels**.** Reproduced from [158], with permission from Springer Nature, 2022.

Compressive Modulus (kPa)	Tensile Modulus (kPa)	Elongation at Break (%)
32.6 ± 3.2	27.5 ± 2.7	63.6 ± 4.1

## Data Availability

Not applicable.
